# Divergence and aggregation of ESG ratings: A survey

**DOI:** 10.12688/openreseurope.19238.1

**Published:** 2025-01-30

**Authors:** Arianna Agosto, Alessandra Tanda

**Affiliations:** 1 Department of Economics and Management, University of Pavia, Pavia, Lombardia, 27100, Italy

**Keywords:** ESG ratings, divergence, aggregation, survey

## Abstract

**Purpose:**

This paper reviews the existing literature on the divergence of Environmental, Social, and Governance (ESG) ratings and explores various aggregation methods. It highlights the challenges posed by inconsistent ESG ratings and their implications for investment decisions.

**Design/methodology/approach:**

The study conducts a comprehensive review of prior research focusing on ESG ratings, examining their correlation levels and the methodologies employed to assess corporate sustainability. It also investigates traditional aggregation techniques and modern machine learning approaches used to address these inconsistencies.

**Findings:**

Past literature reveals that ESG ratings exhibit a low level of correlation across different providers, raising concerns about their reliability as investment indicators. Although some studies propose advanced aggregation methods to enhance accuracy, significant gaps remain in understanding how to effectively consolidate ESG information to create a dependable sustainability indicator.

**Originality:**

This paper provides a critical analysis of the current state of ESG rating methodologies, emphasizing the need for improved aggregation strategies. It underscores the importance of future research in leveraging ESG data to develop more consistent and reliable measures of corporate sustainability.

## 1 Introduction and background

The environmental impact of corporate activities has become increasingly important over the last decades ( Yu *et al.*, 2021). Indeed, companies are made accountable for the impact of their business activities on the planet and society through the measurement of their Corporate Social Performance (CSP).

Many companies have implemented over time sustainability reporting that started initially on a voluntary basis (e.g., the Global Reporting Initiative - GRI) ( Monteiro *et al.*, 2023, for a review) and was later incorporated into the regulatory provisions. For instance, in Europe, the Non-Financial Reporting Directive (NFRD) (Directive 2014/95/EU) introduced in 2014 the requirement for large companies and public-interest entities with more than 500 employees to publish non-financial information related to ESG topics. The directive was later improved and refined by the Corporate Sustainability Reporting Directive (CSRD) (Directive 2022/2464/EU). The new rules expand the NFRD, with the objective to improve the quality and consistency of information provided by companies on the topic of sustainability. Additionally, it enlarges the applicability of these rules to all large companies and, gradually, to listed SMEs. The Directive also introduces European Sustainability Reporting Standards and the external assurance (verification) of sustainability information.

Beside the corporate disclosure, several data providers have started to distribute sustainability scores or ratings, often proxied through the ESG profile of the companies ( Pollman, 2022). This information, together with companies’ disclosure in annual reports or sustainability reports complements financial information available on the market and enables investors to assess the sustainability of corporates, i.e., considering how companies’ behaviour affects external stakeholders and paying attention to the economic, environmental, social, and time factors ( Lozano, 2012; Lozano, 2018; Muñoz-Torres *et al.*, 2019). In other words, this set of information contributes to the reduction of asymmetries of information for investors and, at the same time, a reduction in the cost of capital for corporates ( Yu *et al.*, 2021).

Despite these efforts to promote the diffusion of sustainability disclosure, the birth of multiple ESG ratings providers has brought to a proliferation of methods and scores that often disagree and create “aggregate confusion” on the market as discussed by Berg *et al.* (2022). This has the perverse effect of generating additional uncertainty on the market, rather than actually reducing asymmetries of information for investors.

Motivated by such considerations, the present paper provides a review of the financial and statistical literature concerning the analysis of ESG scores’ divergence and the development of methodologies for ESG ratings/scores aggregations.

The paper is structured as follows: Section 2 provides a brief overview of the ESG ratings and scores; Section 3 motivates the need for developing ESG aggregating measures by presenting the relevant literature focused on ESG rating divergence and its possible sources; Section 4 presents the most relevant contributions in terms of ESG rating aggregation; Section 5 concludes.

## 2 ESG ratings and scores

The ESG profile of companies is often taken as a proxy of their sustainability behaviour ( Eng *et al.*, 2022).

While Environment factors relate to the impact on the environment deriving from the production of goods or services, such as carbon emissions, preservation of the natural environment, biodiversity protection, waste and water management ( European Commission, nd; Robeco, nd; Times, nd), Social factors refer to the impact of the company on society, including issues of employee satisfaction, diversity, inequality, gender gap, protection of young and children, investment in human capital and communities, and human rights ( European Commission, nd; Van Duuren *et al.*, 2016). Governance factors instead are accounted for to evaluate the “good” governance of companies, which can contribute to a more sustainable and balanced firms’ growth, and thus to a more sustainable economic development ( Adams & Mehran, 2012; Alkaraan, 2023; Esteban-Sanchez *et al.*, 2017).

These three factors have also become the basis for investment decisions within sustainable financing and can drive the choice of investors in terms of which companies to finance through equity or debt. Furthermore, since International authorities have started to include sustainability in their agenda ( European Commission, 2018; European Commission, 2021; UN, 2015), policymakers and regulators have developed specific measures. In the financial context, European regulators request financial intermediaries to include ESG aspects for lending and investment activities ( EBA, 2020; ESMA, 2020a; ESMA, 2020b) and call for the inclusion of ESG aspects into creditworthiness evaluations provided by credit rating agencies (European Action Plan for Financing Sustainable Growth) ( European Commission, 2018), to direct funds to the best-performing companies in terms of environmental and social impact. The ESG factors have also a link with economic performance. Events related to climate change can affect profitability and produce losses that can affect companies’ ability to meet their credit obligations. Companies are therefore pushed by different actors towards a more sustainable behaviour. In addition, they are pushed to disclose more about their ESG behaviour, to reduce asymmetries of information and improve their ability to retrieve cheaper funds ( Yu *et al.*, 2021).

To inform the investors concerning corporate ESG performance, specialised companies (including rating agencies) provide measures and proxies for ESG behaviour, publishing ESG ratings or scores that should express the level of sustainability and the degree of accountability of companies on ESG aspects ( Avetisyan & Ferrary, 2013; Scalet & Kelly, 2010). As each rating provider collects information from different sources (company reports, news, stock exchange information, etc.) and applies proprietary methodologies to combine available inputs and produce a synthetic measure of ESG behaviour, ESG evaluations assigned by different providers often produce divergent results ( Abhayawansa & Tyagi, 2021; Berg *et al.*, 2022; Billio *et al.*, 2021; Dimson *et al.*, 2020; Dorfleitner *et al.*, 2015), yielding to potential “sustainability arbitrage” as recently discussed in Pollman (2022). Indeed, multiple ESG ratings for a given company can differ, providing opaque information about the company’s ESG profile. A recent survey by KPMG showed the existence of more than 160 ESG ratings and data providers ( KPMG, 2020), with numerous agencies (eg. Bloomberg, Thomson Reuters, S&P, etc.) whose ESG ratings may differ. The potential presence of divergent scores for the same companies can represent an element of uncertainty for investors and regulators, who may need to choose among different measures and/or combining them into a single metric.

## 3 Divergence of ESG scores

Several recent works showed poor convergence between ESG ratings provided by different agencies and analysed the sources of such inconsistencies. Indeed, understanding the origin of ESG rating divergence can help:

- regulators to put in place proper measures to increase and harmonise ESG disclosure of companies;
- investors to choose between alternative rating providers or to manage divergence through the aggregation of scores issued by different providers;
- companies to improve disclosure of their ESG approach.

Several works showed little convergence between different ESG ratings through empirical studies. Among them, Dorfleitner *et al.* (2015), analysing ESG rating data of publicly traded companies provided by three agencies in the 2002–2012 period, find that the ratings assigned by the considered providers exhibit low linear correlations and significantly differ in distribution, with a small percentage of companies assigned to the same score quartile. The differences in the scoring approaches - revealed in the qualitative evaluation of the three rating methodologies performed in the same paper - partially explain such inconsistencies. Similar conclusions were reached by Chatterji *et al.* (2016), who documented the lack of agreement across ESG ratings issued by six providers between 2002 and 2010, even after adjusting for explicit differences in the definition of CSP held by the different raters. In their review of the related literature, Abhayawansa and Tyagi (2021) examined the possible causes of the differences in the ESG ratings and rankings produced by different agencies, and found that divergences in ESG scores are mainly attributable to disagreements about the definition, composition and weightings for each of the E, S and G factors and to differences in the methodological approaches. In particular, according to Capizzi *et al.* (2021), the weight component is crucial in explaining ESG rating divergences.

The issue of divergence of ESG ratings and its possible causes were explored in depth by Berg *et al.* (2022), who provided a broad empirical study to document the ESG evaluations’ divergence and identify the factors that contribute to the differences. Specifically, the authors consider data from six different ESG rating providers in 2014, including ESG ratings and the related underlying variables. The latter are grouped by imposing a taxonomy of categories, which is then used to decompose the divergence into contributions of scope, measurement, and weight. By re-estimating ESG ratings based on the identified categories, the authors found that the most important dimensions in driving ESG rating divergence are climate risk management, product safety, corporate governance, corruption, and environmental management system. Further differences among ratings arise when considering the Environmental, Social and Governance dimensions separately. Such discrepancies are connected to the nature and measurement of the three factors. Indeed, while the Environmental impact can be relatively easy to measure, the Social and Governance impacts are related to qualitative aspects, which are more difficult to assess. Muñoz-Torres *et al.* (2019) find that rating agencies, when computing ESG scores, mostly focus on the environmental dimension (e.g. waste reduction, energy consumption, water management).

The sources of divergence of ESG rating were the focus of the work by Billio *et al.* (2021), whose findings revealed a lack of commonality in the ESG score criteria used by prominent agencies, with the consequence that agencies assign even opposite ratings to a given company. Differently from previous works, Billio *et al.* (2021) also investigate the implications that such disagreements may have on ESG portfolios performance. Indeed, the identification of sustainable investments and therefore the choice of the relevant benchmarks depends on the ratings provided by agencies. ESG rating heterogeneity leads to the identification of alternative benchmarks, with the consequence that the impact of ESG profile on asset prices is weak and green investments often show no significant difference in financial performances with respect to non-ESG ones.

Despite some criticisms, evidence shows that rating agencies worked to update their computational methodologies for ESG ratings, although they are not able to entirely capture into ratings ( Escrig-Olmedo *et al.*, 2019; Muñoz-Torres *et al.*, 2019). The impact of ESG rating discordance on financial markets was also studied by Wang *et al.* (2024), who found that low ESG score correlation negatively impacts stock excess return rates. In particular, using Chinese market data, the authors found that the smaller the distance between ESG rating issued by domestic and foreign agencies, the higher the excess returns on stocks.

Overall, the presence of ESG scores in the market can encourage companies to improve their CSP behaviour ( Zeng & Xu, 2019), but it also presents possible drawbacks and these must be tackled to ensure comparability of ESG measures.

Standardisation of ESG metrics is, in fact, of primary importance to enable investors to choose among investment opportunities, to allow companies to take appropriate measures to improve their environmental, social and governance footprint, and to improve the degree of transparency of ESG rating providers, as recently underlined by the International Organization of Securities Commissions ( OICV-IOSCO, 2021).

The importance of ESG metrics is further destined to grow in the future and ESG ratings will influence investors’ decisions, therefore affecting firms’ ability to finance their investment and also their capability to pursue a more sustainable business model. The need for ESG metric standardisation is therefore strongly felt by the market, motivated by the growing importance of sustainable finance ^1^ and understanding how ESG ratings provide information to the market and how these ratings can affect creditworthiness is a major managerial and policy challenge.

## 4 ESG score aggregation

In the economic and financial context, it is frequent to construct indicators as the synthesis of multiple input variables. The weighted mean represents a natural solution, but the estimation of weights can be problematic and their choice can be subjective, strongly affecting the final indicators. The issue is so relevant that the European Commission provided a set of recommendations for the construction of aggregated metrics to be used for policy purposes and indicated the steps to be followed to build composite indicators, including data management, weighting, uncertainty and sensitivity analysis ( European Union and Joint Research Centre, 2008).

Data-driven techniques can help build aggregate measures from several input variables. A method widely used in the statistical literature is Principal Component Analysis (PCA), that aims at creating one or more new factors from a larger set of observed variables, where each component is a linear combination of the original variables and orthogonally independent from the other components. A paper by Gucciardi *et al.* (2024) employs PCA (together with pairwise correlation analysis and OLS) to evaluate the “common factors” driving Environmental scores. The study finds a limited number of common factors underlying the E score of different providers, mainly linked to more quantitative variables (e.g., natural resources use).

An alternative to PCA, allowing to take the temporal dimension into account, is represented by Dynamic Factor Models (DFM), which, similarly to PCA, creates new factors through a system of latent variables and parameters associated with the contribution of each observed variable (see Bitetto *et al.*, 2021). Both PCA and DFM make it possible to assess the role played by each variable, easing the interpretability of results. However, both approaches call for a large enough set of variables (at least 10–15), which is not always available.

The need for synthesising variables in a single indicator also arises in the ESG context, and is related to the empirically found divergence of ESG ratings discussed in Section 3. Indeed, when ESG scores provided by different agencies for a given company are discordant, a possible solution for the investor/regulator is to use an aggregated measure which synthesises them into a single metric. Note that the above-mentioned issue of weighting is also crucial in this situation, as the weight attributed to each rating should not be only easily estimated, but also interpreted, allowing the stakeholders to be aware of the contribution of each indicator to the assessment of the company sustainability profile.

The issue of building ESG aggregated scores was addressed by two recent works. Agosto *et al.* (2023a) and Agosto *et al.* (2023b) relied on a Bayesian approach - based on the methodological results of Giudici *et al.* (2003) and Cerchiello and Giudici (2014) - to obtain an aggregated indicator for the ESG performance of companies by integrating ratings assigned by different providers. In both works the aggregated ESG indicator is computed starting from single ESG scores, by attributing a weight to each. The proposed weighting procedure is data-driven, as it relies on the relationship between the ESG performance and the creditworthiness measured by credit ratings issued by recognised agencies.

In Agosto *et al.* (2023b), each available ESG score receives a weight that is a function of the likelihood of the observed counts of credit rating classes, under the alternative partitions generated by the ESG scores. The likelihood weights are obtained through the application of Bayes’ theorem. The relationship between ESG scores and credit rating was suggested by previous research and prompted by policymakers and regulators ( EBA, 2020; Lagoarde-Segot, 2019). The proposed methodology is applied to a sample of 791 European companies for which the ESG scores issued by three different providers, together with Moody’s credit rating, are available. For each of the mentioned variables, the most recent value in the 2018–2020 period is considered. After estimating the likelihood weights for the considered ESG scores, the authors performed an out-of-sample analysis to check whether the combined score improves the accuracy of credit rating predictions, as measured by using the distance (in notches) between the observed and predicted credit rating class. The findings show that the merged score reduces the overestimation of credit risk (lower positive error) for the bestrated companies (AA, A and BBB classes) with respect to the individual scores, while, for the BB and B-rated companies, the cases of credit risk underestimation (negative errors) are more frequent and higher in magnitude with the combined ESG score than with the individual ones. In other words, according to the results of Agosto *et al.* (2023b), the merged sustainability rating seems to better recognise the most credit-reliable companies.

The Bayesian approach is also used by Agosto *et al.* (2023a), who built an indicator of ESG performance of listed companies that integrates the ESG scores assigned by different providers. In this work, the credit-related target variable used to weight the single scores is a binary one which indicates whether a company’s credit rating is speculative or investment grade, according to the Bloomberg Issuer Default Risk model. Specifically, the higher the capability of the ESG score to classify companies into speculative and investment grade, the higher the attributed weight. The authors apply the described methodology to a training sample of European listed companies and validate the estimated weights on a testing sample. For each company, the probability of belonging to a speculative rating class is calculated as the weighted mean of the probabilities assigned by the three considered scores, using the Bayesian likelihood-based weights. By comparing the classification performance - in terms of Receiving Operating Curve (ROC) - of the aggregated score with that of the individual ones, the authors found that the merged score performs better in the tails of the distribution, where the more extreme financial profiles (very bad or very good companies) lie. The authors also showed that the results are robust to the inclusion of financial ratios as control variables. Bayesian learning techniques are not the only possible solution to statistically aggregate single indicators using predictive accuracy. Indeed, Agosto *et al.* (2023a) also showed the application of an artificial intelligence method, XGBOOST ( Chen, 2015), an ensemble model which works over the idea of combining several weak classifiers to create a strong one characterised by extremely good performance thanks to a regularised gradient boosting framework. The XGBOOST and the Bayesian model lead to very similar results in terms of classification performance, but, as claimed by the authors, the latter provides explainable weights which can be interpreted and used in further evaluations.

## 5 Concluding remarks

This paper has reviewed the literature on the divergence of ESG ratings. It has become evident that the proliferation of ESG rating providers has brought a high number of ratings that investors, policymakers and markets can access to evaluate the sustainability of companies. While this can be positive, there is a general lack of consensus on the ”best” rating to be used as these ratings have a high level of divergence. Hence, this can create confusion and have the perverse effect of increasing uncertainty instead of reducing asymmetries of information. To partially overcome this issue, a few studies have tried to employ statistical aggregation techniques to lever the information included in each rating and provide more coherent and complete information on the overall ESG profile of corporates. Studies employ both traditional techniques (e.g., PCA or OLS) and more advanced algorithms (e.g., XGBOOST). There is, to date, no strictly preferable approach and further research is needed to provide additional insights into the divergence of ESG ratings and new attempts are needed to improve the utility and accuracy of aggregation methods. For sure, the definition of a harmonised regulatory framework and an increase in the transparency of these ratings will help to solve this divergence. Also, from the corporates’ point of view, understanding what are the drivers of ESG ratings will help them to better plan their strategies to improve their environmental, social and governance performance in an effective way that produces a tangible effect on their performance or risk and hence that determines their ability to stay on the market.

## Ethics and consent

Ethical approval and consent were not required.

## Data Availability

No data are associated with this article.

## Footnotes

^1^ Just as a background information, the number of sustainable investment funds available in Europe grew from 200 at the end of 2016 to 3,196 at the end of 2020 ( Morningstar, 2021), with a corresponding growth in the managed assets.
