# Divergence and aggregation of ESG ratings: A survey

**DOI:** 10.12688/openreseurope.19238.2

**Published:** 2025-06-30

**Authors:** Arianna Agosto, Alessandra Tanda

**Affiliations:** 1 Department of Economics and Management, University of Pavia, Pavia, Lombardia, 27100, Italy

**Keywords:** ESG ratings, divergence, aggregation, survey

## Abstract

**Purpose:**

This paper reviews the existing literature on Environmental, Social, and Governance (ESG) ratings divergence and aggregation methods. It highlights the challenges posed by inconsistent ESG ratings and their implications for investment decisions.

**Design/methodology/approach:**

The study conducts a comprehensive review of prior research focusing on ESG ratings, examining their correlation levels and the methodologies employed to assess corporate sustainability. It also investigates traditional aggregation techniques and modern machine learning approaches used to address these inconsistencies.

**Findings:**

The review reveals that ESG ratings exhibit a low level of correlation across different providers, raising concerns about their reliability as investment indicators. Although some studies propose advanced aggregation methods to enhance accuracy, significant gaps remain in understanding how to effectively consolidate ESG information to create a dependable sustainability indicator.

**Originality:**

This paper provides a critical analysis of the current state of ESG rating methodologies, emphasizing the need for improved aggregation strategies. It underscores the importance of future research in leveraging ESG data to develop more consistent and reliable measures of corporate sustainability.

## 1 Introduction and background

The environmental impact of corporate activities has become increasingly important over the last decades ( Yu *et al.*, 2021). Indeed, companies are made accountable for the impact of their business activities on the planet and society through the measurement of their Corporate Social Performance (CSP).

Many companies have implemented over time sustainability reporting that started on a voluntary basis (e.g., the Global Reporting Initiative - GRI) ( Monteiro *et al.*, 2023, for a review) and was later incorporated into regulatory provisions. For instance, in Europe, the Non-Financial Reporting Directive (NFRD) (Directive 2014/95/EU) introduced in 2014 the requirement to publish non-financial information related to ESG topics for large companies and public-interest entities with more than 500 employees. The directive was then revised by the Corporate Sustainability Reporting Directive (CSRD) (Directive 2022/2464/EU). The new rules expand the NFRD, to improve the quality and consistency of information provided by companies on sustainability. Additionally, it enlarges the applicability of these rules to all large companies and, gradually, to listed SMEs. The Directive also introduces European Sustainability Reporting Standards and the external assurance (verification) of sustainability information.

Besides corporate disclosure requirements, several data providers developed sustainability scores or ratings, often proxied through the ESG profile of the companies ( Pollman, 2022). This information, together with companies’ disclosure in annual reports or sustainability reports complements financial information available on the market and enables investors to assess the sustainability of corporates, i.e., considering how companies’ behaviour affects external stakeholders and paying attention to the economic, environmental, social, and time factors ( Lozano, 2012; Lozano, 2018; Muñoz-Torres *et al.*, 2019). In other words, this set of information contributes to the reduction of asymmetries of information for investors and can determine a reduction in the cost of capital for corporates ( Yu *et al.*, 2021).

Despite these efforts to promote the diffusion of sustainability disclosure, the existence of multiple ESG rating providers determined to a proliferation of methods and scores that often disagree and create “aggregate confusion” on the market ( Berg *et al*., 2022). This has the perverse effect to generate additional uncertainty, rather than actually reducing asymmetries of information on the market for investors.

This paper surveys the evidence on ESG rating divergence: low correlation between ESG ratings of different providers is determined by differences in methods and definitions. Low agreement impacts the investment strategies and outcomes of portfolio allocation ( Erhart, 2022).

Understanding the origin of ESG rating divergence can help regulators put in place proper measures to increase and harmonise the ESG disclosure of companies, investors to choose between alternative rating providers or to manage divergence through the aggregation of scores issued by different providers, and companies to improve disclosure of their ESG approach

The paper also reviews the existing methods to overcome divergence by aggregating different ESG scores. Traditional techniques and more advanced approaches coexist in the literature, with their advantages and limits.

The manuscript contributes to the discussion on ESG rating transparency and harmonisation and formulates research questions that warrant attention from scholars.

The paper is structured as follows: Section 2 provides a brief overview of the ESG ratings and scores; Section 3 motivates the need for developing ESG aggregating measures by presenting the relevant literature focused on ESG rating divergence and its possible sources; Section 4 presents the most relevant contributions in terms of ESG rating aggregation; Section 5 concludes.

## 2 ESG ratings and scores

The ESG profile of companies is often taken as a proxy of their sustainability behaviour ( Eng *et al.*, 2022).

While Environment factors relate to the impact on the environment deriving from the production of goods or services, such as carbon emissions, preservation of the natural environment, biodiversity protection, waste and water management ( European Commission, nd; Robeco, nd; Times, nd); Social factors measure the impact of the company on society, including issues of employee satisfaction, diversity, inequality, gender gap, protection of young and children, investment in human capital and communities, and human rights ( European Commission, nd; Van Duuren *et al.*, 2016); Governance factors evaluate the “good” governance of companies, which can contribute to a more sustainable and balanced firms’ growth, and thus to a more sustainable economic development ( Adams & Mehran, 2012; Alkaraan, 2023; Esteban-Sanchez *et al.*, 2017).

These three factors have also become the basis for investment decisions within sustainable financing and can drive the choice of investors in terms of which companies to finance through equity or debt. Furthermore, since international authorities have included sustainability in their agenda ( European Commission, 2018; European Commission, 2021; UN, 2015), policymakers and regulators have developed specific provisions. In the financial market, European regulators request financial intermediaries to include ESG aspects for lending and investment decisions ( EBA, 2020; ESMA, 2020a; ESMA, 2020b) and request credit rating agencies to include ESG aspects into creditworthiness evaluations (European Action Plan for Financing Sustainable Growth) ( European Commission, 2018). The aim is increase allocative efficiency and direct funds to the best-performing companies in terms of environmental and social impact.

The ESG factors are also related to economic performance. Events caused by climate change can affect profitability and produce losses able to affect companies’ ability to meet their credit obligations. Companies are therefore pushed by different actors towards a more sustainable behaviour. In addition, they are incentivised to disclose more about their ESG behaviour, to reduce asymmetries of information and improve their ability to retrieve cheaper funds ( Yu *et al.*, 2021).

To inform investors concerning corporate ESG performance, specialised companies (including rating agencies) publish ESG ratings or scores that express the level of sustainability and the degree of accountability of companies on ESG aspects ( Avetisyan & Ferrary, 2013; Scalet & Kelly, 2010). Each rating provider collects information from different sources (company reports, news, stock exchange information, etc.) and applies proprietary methodologies to combine available inputs and produce a synthetic measure of ESG behaviour.

ESG evaluations assigned by different providers often produce divergent results ( Abhayawansa & Tyagi, 2021; Berg *et al.*, 2022; Billio *et al.*, 2021; Dimson *et al.*, 2020; Dorfleitner *et al.*, 2015), yielding to potential “sustainability arbitrage” ( Pollman, 2022). Indeed, divergent ESG ratings for a given company may emerge, providing opaque information about the company’s ESG profile and pave the way for greenwashing practices ( Agnese *et al.*, 2025). KPMG surveyed more than 160 ESG ratings and data providers ( KPMG, 2020), with numerous agencies (eg. Bloomberg, Thomson Reuters, S&P, etc.) and found their ESG ratings are divergent. The outcome and impact dimensions of ESG behaviour have limited effect in determining ESG ratings ( Guerrero & Viteri, 2025).

The presence of divergent scores for the same companies increases uncertainty for investors and regulators, who may need to choose among different measures and/or combine them into a single metric.

As the importance of ESG metrics will grow in the future and influence investors’ decisions, and firms’ ability to finance their investment, the market feels a strong need for ESG metric standardisation. This represents a major managerial and policy challenge, coupled with the growing importance of sustainable finance ^1^ and climate change effects on creditworthiness of companies.

## 3 Methods

We searched the database Scopus for manuscripts on ESG ratings divergence and aggregation in early April 2025. We employed the following search strings for divergence (TITLE-ABS-KEY (("ESG score*" OR "ESG RATING*" ) AND "*AGREE*" ) OR TITLE-ABS-KEY (("ESG score*" OR "ESG RATING*" ) AND "*VERGE*" ) ) and for aggregation TITLE-ABS-KEY (("ESG score*" OR "ESG RATING*" ) AND "*aggr*" ).

The search returned, respectively, 173 and 90 documents. We manually scanned through the titles, abstracts and keywords and selected the papers that explicitly discuss divergence of ESG ratings, the consequences on financial markets and investors, and aggregation techniques. We eliminate irrelevant studies and review the remaining to provide a bird’s eye view of the literature available to researchers approaching this topic.

## 4 Divergence of ESG scores

Empirical evidence shows little convergence between different ESG ratings through empirical studies ( Anselmi & Petrella, 2025; Bissoondoyal-Bheenick *et al.*, 2024; Vasiu, 2024).

In most works, the disagreement in ESG evaluations is expressed in terms of low pairwise correlations between the ESG ratings of different providers. As ESG scores are usually continuous, the most natural choice to model their correlation is the Pearson correlation coefficient.

Specifically, given two providers, *i* and *j*, each of which assigning ESG scores to a set of *N* companies, the Pearson correlation coefficient between the scores assigned by *i* and *j* is calculated as:


$$\rho_{ij}^{P}=\frac{\sigma_{ij}}{\sigma_{i}\sigma_{j}}$$

Where *σ _ij_* is the covariance between ESG scores *i* and *j*, calculated on the *N*-sized sample of companies, while *σ _i_* and *σ _j_* are the standard deviations of scores *i* and *j* respectively.

Pearson correlation is also used by Bissoondoyal-Bheenick *et al.* (2024) to evaluate the similarity of ESG scores aggregated with different methods (see Section 5).

However, correlation can be also calculated through rank-based measures such as the Spearman coefficient, defined as:


$$\rho_{ij}^{S}=\frac{\sigma_{ij}^{R}}{\sigma_{i}^{R}\sigma_{j}^{R}}$$

where the $\sigma_{ij}^{R}$ covariance and the $\sigma_{i}^{R}$ and $\sigma_{j}^{R}$ standard deviations are calculated using the scores converted to ranks, rather than the original ones.

Rank correlation is used by Billio *et al.* (2021), together with mean absolute error and the percentage of observed agreement among the considered rating providers.

Among the studies focused on ESG score divergence, Dorfleitner *et al.* (2015), analysing ESG rating data of publicly traded companies provided by three agencies in the 2002–2012 period, find that the ratings assigned by the considered providers exhibit low linear correlations and significantly differ in distribution, with a small percentage of companies assigned to the same score quartile. The differences in the scoring approaches - revealed in the qualitative evaluation of the three rating methodologies performed in the same paper - partially explain such inconsistencies. Similar conclusions were reached by Chatterji *et al.* (2016), who documented the lack of agreement across ESG ratings issued by six providers between 2002 and 2010, even after adjusting for explicit differences in the definition of CSP held by the different raters. In their review of the related literature, Abhayawansa and Tyagi (2021) examined the possible causes of the differences in the ESG ratings and rankings produced by different agencies, and found that divergences in ESG scores are mainly attributable to disagreements about the definition, composition and weightings for each of the E, S and G factors and to differences in the methodological approaches. In particular, according to Capizzi *et al.* (2021), the weight component is crucial in explaining ESG rating divergences. Bissoondoyal-Bheenick *et al.* (2024) find high correlations between Asset4 and Sustainalytics, but not among other providers.

The issue of divergence of ESG ratings and its possible causes were explored in depth by Berg *et al.* (2022), who provided a broad empirical study to document the ESG evaluations’ divergence and identify the factors that contribute to the differences. Specifically, the authors consider data from six different ESG rating providers in 2014, including ESG ratings and the related underlying variables. The latter are grouped by imposing a taxonomy of categories, which is then used to decompose the divergence into contributions of scope, measurement, and weight. By re-estimating ESG ratings based on the identified categories, the authors found that the most important dimensions in driving ESG rating divergence are climate risk management, product safety, corporate governance, corruption, and environmental management system. Further differences among ratings arise when considering the Environmental, Social and Governance dimensions separately. Such discrepancies are connected to the nature and measurement of the three factors. Indeed, while the Environmental impact can be relatively easy to measure, the Social and Governance impacts are related to qualitative aspects, which are more difficult to assess. Muñoz-Torres *et al.* (2019) find that rating agencies, when computing ESG scores, mostly focus on the environmental dimension (e.g. waste reduction, energy consumption, water management).

The sources of divergence of ESG rating consist of a lack of commonality in the ESG score criteria used by prominent agencies, with the consequence that agencies assign even opposite ratings to a given company ( Billio *et al.*, 2021). Kurbus and Rant (2025) discuss the legal drivers of ESG ratings disagreement, differentiating between civil law and common law firms: correlation disagreement is lower for civil law firms, while dispersion disagreement is low for common law corporates. Also country and company characteristics can influence the ESG ratings disagreement ( Rubino *et al.*, 2024). Despite some criticisms, evidence shows that rating agencies worked to update their computational methodologies for ESG ratings, although they are not able to entirely capture into ratings ( Escrig-Olmedo *et al.*, 2019; Muñoz-Torres *et al.*, 2019).

Many studies focus on the impact of ESG divergence on corporate performance, market efficiency, and portfolio decisions and performance. ESG score disclosure can encourage companies to improve their CSP behaviour ( Zeng & Xu, 2019), but ESG disagreement presents possible drawbacks. Firms tend to reduce corporate green innovation ( Li *et al.*, 2024; Li *et al.*, 2025; Xiao *et al.*, 2025; Yuan *et al.*, 2024; Zhu *et al.*, 2025) or total factor productivity ( Li & Yang, 2025) when ESG ratings disagree. But ESG rating divergence can result in additional efforts by companies to promote the quality and quantity of green innovation ( Wan *et al.*, 2025; Zhou *et al.*, 2024; Zhou *et al.*, 2025). ESG rating disagreement can also act as a moderating factor for the corporate performance, e.g., efficiency ( Lin *et al.*, 2025). 

Billio *et al.* (2021) investigate the implications that ESG rating disagreements may have on ESG portfolios performance. Indeed, the identification of sustainable investments and therefore the choice of the relevant benchmarks depends on the ratings provided by agencies. ESG rating heterogeneity leads to the identification of alternative benchmarks, with the consequence that the impact of ESG profile on asset prices is weak and green investments often show no significant difference in financial performances with respect to non-ESG ones.

ESG rating disagreement improves stock pricing efficiency ( Yin *et al.*, 2025), also under high analysts’ attention ( Tan *et al.*, 2025), but contradicting evidence highlights the benefits of reducing investor ambiguity via an improved market efficiency ( Bachner, 2025).

ESG rating discordance also negatively impacts stock excess return rates; using Chinese market data, Wang *et al.* (2024) found that the smaller the distance between ESG rating issued by domestic and foreign agencies, the higher the excess returns on stocks.

## 5 ESG score aggregation

In the economic and financial context, scholars frequently construct indicators to synthesise multiple input variables. The need for synthesising variables in a single indicator also arises in the ESG context and is related to the empirically found divergence of ESG ratings discussed in Section 3. Indeed, when ESG scores provided by different agencies for a given company are discordant, a possible solution for the investor/regulator is to use an aggregated measure which synthesises them into a single metric.

The recent academic literature provides ways to correct the divergence in ESG ratings ( Dupuy & Garibal, 2022) and solutions to build ESG-aggregated scores.

The weighted mean represents a simple and reliable solution ( Bissoondoyal-Bheenick *et al.*, 2024), but the estimation of weights can be problematic and their choice can be subjective, affecting the final indicators. Indeed, the most natural mathematical formulation for an aggregated ESG indicator $\left(ESG_{aggr}^{i}\right)$ is a linear combination of the scores assigned to the same company *i* by a set of *k* = 1,..., *K* providers $\left(ESG_{k}^{i}\right)$ and the corresponding *w _k_* weights :


$$ESG_{aggr}^{i}=\sum_{k=1}^{K}w_{k}ESG_{k}^{i}$$

However, the estimation of weights can be problematic, and their choice can be subjective, affecting the final indicators. The issue is so relevant that the European Commission provided a set of recommendations for the construction of aggregated metrics to be used for policy purposes and indicated the steps to be followed to build composite indicators, including data management, weighting, uncertainty and sensitivity analysis ( European Union and Joint Research Centre, 2008). Erhart (2022) refines the weighted mean approach using Montecarlo simulations and varying weights (1/N ±25%) to build an aggregate ESG score by the arithmetic, geometric, or harmonic mean.

Data-driven techniques can help build aggregate measures from several input variables. A method widely used in the statistical literature is Principal Component Analysis (PCA), that aims at creating one or more new factors from a larger set of observed variables, where each component is a linear combination of the original variables and orthogonally independent from the other components. Gucciardi *et al.* (2024) employ PCA (together with pairwise correlation analysis and OLS) to evaluate the “common factors” driving Environmental scores. The study finds a limited number of common factors underlying the E score of different providers, mainly linked to more quantitative variables (e.g., natural resources use). Bissoondoyal-Bheenick *et al.* (2024) apply PCA and voting average to Sustainalytics, MSCI, and Asset4 ESG ratings and conclude the methods might contribute to ESG rating divergence.

The limits of PCA emerge when building indexes with a normative value, according to Gai *et al.* (2023), who developed an alternative aggregation method based on Multidimensional Synthesis of Indicators (MSI). The authors claim this method can also help in detecting greenwashing practices in companies. Dynamic Factor Models (DFM) also represent an alternative to PCA that allows taking the temporal dimension into account. Similarly to PCA, DFM creates new factors through a system of latent variables and parameters associated with the contribution of each observed variable (see Bitetto *et al.*, 2021). Both PCA and DFM assess the role played by each variable, easing the interpretability of results. However, both approaches require a large set of variables (at least 10–15), which is not always available. Note that the above-mentioned issue of weighting is also crucial in this situation, as the weight attributed to each rating should not only be easily estimated, but also interpreted, allowing the stakeholders to be aware of the contribution of each indicator to the assessment of the company sustainability profile.

Agosto *et al.* (2023a) and Agosto *et al.* (2023b) relied on a Bayesian approach - based on Giudici *et al.* (2003) and Cerchiello and Giudici (2014) - to obtain an aggregated indicator for the ESG performance of companies by integrating ratings assigned by different providers. In both works the aggregated ESG indicator is computed starting from single ESG scores, by attributing a weight to each. The proposed weighting procedure is data-driven, as it relies on the relationship between the ESG performance and the creditworthiness measured by credit ratings issued by recognised agencies.

In Agosto *et al.* (2023b), each available ESG score receives a weight that is a function of the likelihood of the observed counts of credit rating classes, under the alternative partitions generated by the ESG scores. The likelihood weights are obtained through the application of Bayes’ theorem. The relationship between ESG scores and credit rating was suggested by previous research and prompted by policymakers and regulators ( EBA, 2020; Lagoarde-Segot, 2019). The proposed methodology is applied to a sample of 791 European companies for which the ESG scores issued by three different providers, together with Moody’s credit rating, are available. For each of the mentioned variables, the most recent value in the 2018–2020 period is considered. After estimating the likelihood weights for the considered ESG scores, the authors performed an out-of-sample analysis to check whether the combined score improves the accuracy of credit rating predictions, as measured by using the distance (in notches) between the observed and predicted credit rating class. The findings show that the merged score reduces the overestimation of credit risk (lower positive error) for the best rated companies (AA, A and BBB classes) with respect to the individual scores, while, for the BB and B-rated companies, the cases of credit risk underestimation (negative errors) are more frequent and higher in magnitude with the combined ESG score than with the individual ones. In other words, according to the results of Agosto *et al.* (2023b), the merged sustainability rating seems to better recognise the most credit-reliable companies.

The Bayesian approach is also used by Agosto *et al.* (2023a), who built an indicator of ESG performance of listed companies that integrates the ESG scores assigned by different providers. In this work, the credit-related target variable used to weight the single scores is a binary one which indicates whether a company’s credit rating is speculative or investment grade, according to the Bloomberg Issuer Default Risk model. Specifically, the higher the capability of the ESG score to classify companies into speculative and investment grade, the higher the attributed weight. The authors apply the described methodology to a training sample of European listed companies and validate the estimated weights on a testing sample. For each company, the probability of belonging to a speculative rating class is calculated as the weighted mean of the probabilities assigned by the three considered scores, using the Bayesian likelihood-based weights. By comparing the classification performance - in terms of Receiving Operating Curve (ROC) - of the aggregated score with that of the individual ones, the authors found that the merged score performs better in the tails of the distribution, where the more extreme financial profiles (very bad or very good companies) lie. The authors also showed that the results are robust to the inclusion of financial ratios as control variables.

Besides Bayesian learning techniques, machine learning and AI algorithms are used to statistically aggregate single indicators. Tech-driven alternative methods provide a more transparent and democratic aggregation process ( Hughes *et al.*, 2021). Indeed, Agosto *et al.* (2023a) applied an artificial intelligence method, XGBOOST ( Chen, 2015), an AI ensemble model which works over the idea of combining several weak classifiers to create a strong one characterised by extremely good performance thanks to a regularised gradient boosting framework. The XGBOOST and the Bayesian model lead to very similar results in terms of classification performance, but, as claimed by the authors, the latter provides explainable weights which can be interpreted and used in further evaluations. Indeed, when, as in this case, a machine learning technique not explainable by design leads to a poor gain in predictive accuracy, the use of computationally expensive AI methods like Shapley values is not justified. López-García *et al.* (2025) apply Un-weighted TOPSIS - technique for order preference by similarity to ideal solution - to Top 100 EMEA companies and provide an ESG ranking of companies that solves the divergence in ratings.

It is worth mentioning that, regardless of the specific aggregation methodology, it is difficult to measure and compare the performance of the obtained ESG indicators, due to the absence of a target variable. Indeed, differently from credit scoring models, which are validated using the actual incidence of defaults, the ESG behaviour cannot be easily and objectively summarised by an observed variable. Indeed, among the presented works, some use unsupervised techniques such as PCA ( Bissoondoyal-Bheenick *et al.*, 2024; Gucciardi *et al.*, 2024), while others rely on the relationship between ESG and financial performance ( Billio *et al.*, 2021; Gai *et al.*, 2023) or credit worthiness measured by agency ratings ( Agosto *et al.*, 2023a; Agosto *et al.*, 2023b).

## 6 Concluding remarks

This paper has reviewed the literature on the divergence of ESG ratings. It has become evident that the proliferation of ESG rating providers has brought a high number of ratings that investors, policymakers and markets can access to evaluate the sustainability of companies. While this can be positive, there is a general lack of consensus on the ”best” rating to be used as these ratings have a high level of divergence. Hence, this can create confusion and have the perverse effect of increasing uncertainty instead of reducing asymmetries of information.

To partially overcome this issue, a few studies have tried to employ statistical aggregation techniques to leverage the information included in each rating and provide more coherent and complete information on the overall ESG profile of firms. Studies employ both traditional techniques (e.g., PCA or OLS) and more advanced algorithms (e.g., XGBOOST). There is, to date, no strictly preferable approach and further research is needed to provide additional insights into the divergence of ESG ratings and new attempts are needed to improve the utility and accuracy of aggregation methods. In addition, the unavailability of an homogeneous and easily implementable testing framework represents a limitation of the existing ESG aggregation methodologies. Applying cluster analysis to the proposed ESG scores and identifying common financial and strategic features of companies belonging to the same clusters is one of the aspects that deserve further research, together with a more in- depth analysis of the topic of explainability of the weights attributed to different scores.

For sure, the definition of a harmonised regulatory framework and an increase in the transparency of these ratings will help to solve ESG score divergence. Also, from the corporates’ point of view, understanding what are the drivers of ESG ratings will help them to better plan their strategies to improve their environmental, social and governance performance in an effective way that produces a tangible effect on their performance or risk and hence that determines their ability to stay on the market.

Policy efforts to improve standardisation of ESG metrics remains vital for investors to choose among investment opportunities; companies to take appropriate measures to improve their environmental, social and governance footprint; and ESG rating providers to improve their transparency ( OICV-IOSCO, 2021).

## Ethics and consent

Ethical approval and consent were not required.

## Data Availability

No data are associated with this article.

## Footnotes

^1^ Just as a background information, the number of sustainable investment funds available in Europe grew from 200 at the end of 2016 to 3,196 at the end of 2020 ( Morningstar, 2021), with a corresponding growth in the managed assets.
