# Supplementary material for: Divergence and aggregation of ESG ratings: A survey
Source: Open Res Eur. 2025 Jun 30;5:28. Originally published 2025 Jan 30. [Version 2] doi: 10.12688/openreseurope.19238.2 (PMC12398680; doi:10.12688/openreseurope.19238.2)
Supplement: Peer review history

# Reviewer report: Reviewer response for version 2

**DOI:** 10.21956/openreseurope.22316.r56291

**Published:** 2025-09-18

**Authors:** Luca Di Persio

**Affiliations:** 1 Verona University, Verona, Italy

**To the Editorial Office**

**Subject**: Final Assessment of Revised Manuscript “Divergence and Aggregation of ESG Ratings: A Survey”

 Dear Editors,

  I am writing to convey my final judgment regarding the revised (second version) manuscript entitled *“Divergence and Aggregation of ESG Ratings: A Survey”* by Arianna Agosto and Alessandra Tanda. After carefully reviewing this amended version, I am pleased to recommend the acceptance of the paper for indexing in its current form.

 Indeed, the authors have undertaken a commendable effort to address my (previously written, composing the first paper's review) comments and substantially improve the manuscript. In particular, the authors have significantly strengthened the introduction by offering a clearer articulation of the motivation behind the study and the central contributions it makes to the literature on ESG score divergence and aggregation. Moreover, a new, dedicated section outlines the methodology adopted to select and screen the literature under review. The latter enhances the transparency and reproducibility of the research process. I would also like to underline that Section 5 has been expanded to offer a more thorough discussion of the metrics used to evaluate divergence among ESG ratings. The authors provide a clear exposition of correlation-based methods, such as Pearson and Spearman coefficients, and have incorporated recent empirical studies applying unsupervised learning methods like Principal Component Analysis (PCA). Analogously, They have directly acknowledged the persistent absence of a standardised framework for evaluating ESG aggregation methods, hence addressing a critical limitation in the current literature. It is also worth to mention that the concluding section has been extended to propose several promising avenues for future investigation. These include the use of clustering techniques for ESG profile classification and the exploration of interpretability tools, such as Shapley values, to evaluate aggregation weights. Importantly, the authors critically assess the trade-offs between methodological complexity and practical utility.

 Summing up, the paper now offers a timely and complete contribution to a field of increasing practical and theoretical importance. The Authors systematically review a broad and up-to-date body of literature and succeed in synthesizing a wide range of methodological approaches for ESG score aggregation, moreover, by highlighting methodological discrepancies across ESG providers and proposing pathways for methodological convergence, the paper has also increased its relevance for both academic researchers and market practitioners.

 For all what above, I recommend the indexing of the submitted, amended version of the article without further revision.

 Sincerely,

  Luca Di Persio

  College of Mathematics - Dept. Comp.Science.

  University of Verona

Are all factual statements correct and adequately supported by citations?

Yes

Is the review written in accessible language?

Yes

Are the conclusions drawn appropriate in the context of the current research literature?

Yes

Is the topic of the review discussed comprehensively in the context of the current literature?

Yes

Reviewer Expertise:

Stochastic analysis and applications, financial arguments included. Machine learning and application to time series forecasting, LLM-based models, calibration methodologies.

I confirm that I have read this submission and believe that I have an appropriate level of expertise to confirm that it is of an acceptable scientific standard.

# Reviewer report: Reviewer response for version 2

**DOI:** 10.21956/openreseurope.22316.r56575

**Published:** 2025-08-30

**Authors:** Diana Elena Vasiu

**Affiliations:** 1 Lucian Blaga University of Sibiu, Sibiu, Romania

The paper titled *Divergence and Aggregation of ESG Ratings: A Survey* by Arianna Agosto and Alessandra Tanda provides an extensive literature survey on the divergence of Environmental, Social, and Governance (ESG) and the methodologies proposed for their aggregation.  It emphasizes the challenges that inconsistent ratings across providers create for investors, regulators, and corporations, and critically examines both traditional statistical techniques and contemporary machine learning approaches proposed to address these discrepancies.

 The authors perform an extensive and current literature review, citing over 60 academic and regulatory sources. The review integrates legal, methodological, and economic perspectives, addressing both foundational works and very recent contributions.

 The paper responds to prior peer reviews by improving clarity and adding more recent and relevant studies, expanding the discussions of statistical divergence measures (Pearson, Spearman), clustering, PCA, and AI-based approaches. Furthermore, it emphasised the absence of a standardised and widely accepted framework for evaluating the performance of ESG score aggregation methods.

 The article’s factual assertions are consistently substantiated by peer-reviewed research, official reports, and regulatory documentation, thereby minimizing speculative interpretations and ensuring claims are grounded in credible sources. While the citations are accurate, some referenced studies are novel or rely on proprietary data, and thus their findings may not yet have been independently validated or broadly adopted within the field. Moreover, the literature review places considerable emphasis on European regulatory frameworks, which may not fully capture global perspectives.  Future research would benefit from expanding the literature base to include more diverse geographical and institutional perspectives, thereby enhancing the representativeness and global applicability of the analysis.

 The review is written in a clear and accessible academic style, making it suitable for a multidisciplinary audience across finance, economics, public policy, and sustainability. Although it engages with complex methodologies such as Principal Component Analysis, Bayesian inference, and machine learning, advanced techniques are explained using accurate yet comprehensible language, maintaining analytical rigor while remaining comprehensible to readers without specialized quantitative expertise. But despite efforts to clarify, sections involving advanced statistical or machine learning methods may still present a barrier for readers lacking a strong quantitative background.

 The conclusions presented in the review are closely aligned with the current state of research on ESG rating divergence and aggregation. They accurately synthesize empirical findings and theoretical insights, reflecting the fragmented methodologies and ongoing discrepancies among ESG rating providers. The authors acknowledge the persistent lack of consensus among ESG rating providers, the methodological fragmentation across studies, and the challenges of creating standardized aggregation frameworks. They underscore the need for future research focused on developing explainable, data-driven, and policy-relevant aggregation models. The conclusions are both measured and forward-looking, offering well-grounded directions for further investigation without overgeneralizing findings or making unsupported claims. By stressing the importance of transparency and harmonization, the review’s conclusions are well-positioned to inform ongoing regulatory debates and initiatives.

Are all factual statements correct and adequately supported by citations?

Yes

Is the review written in accessible language?

Yes

Are the conclusions drawn appropriate in the context of the current research literature?

Yes

Is the topic of the review discussed comprehensively in the context of the current literature?

Yes

Reviewer Expertise:

Financial Analysis, Accounting, Fundamentals of statistics, Financial markets, ESG(Environmental, Social, and Governance)

I confirm that I have read this submission and believe that I have an appropriate level of expertise to confirm that it is of an acceptable scientific standard.

# Reviewer report: Reviewer response for version 1

**DOI:** 10.21956/openreseurope.20823.r50578

**Published:** 2025-02-24

**Authors:** Luca Di Persio

**Affiliations:** 1 Verona University, Verona, Italy

The paper titled *Divergence and Aggregation of ESG Ratings: A Survey* by Arianna Agosto and Alessandra Tanda presents an extensive review of the literature concerning the divergence of Environmental, Social, and Governance (ESG) ratings and the methodologies proposed for their aggregation.

 The authors highlight the inconsistencies observed across ESG ratings provided by different agencies, emphasizing the implications of such discrepancies on investment decision-making, regulatory policies, and corporate sustainability strategies. The study adopts a methodological approach grounded in a systematic review of previous works, critically evaluating the statistical and econometric techniques employed to assess and consolidate ESG ratings into a unified indicator.

 The main findings suggest that ESG ratings exhibit a low level of correlation across different providers, which undermines their reliability as a basis for investment strategies. The sources of such divergence can be traced back to variations in scope, measurement techniques, and weighting schemes employed by different agencies. In response to this issue, various aggregation techniques have been proposed, ranging from classical statistical approaches, e.g., Principal Component Analysis (PCA) and Ordinary Least Squares (OLS) regression, to more sophisticated ML-based methods, including Bayesian aggregation and gradient boosting algorithms such as XGBoost. The article underscores that no single method has emerged as universally optimal, thereby necessitating further research into the design of robust aggregation techniques that enhance the reliability of ESG ratings.

 It is worth mentioning that the paper provides a well-structured discussion of ESG rating divergence and its consequences. Nevertheless, certain aspects require further refinement to strengthen its scientific rigour. In particular, I suggest the Authors to better formalise the theoretical framework to structure the discussion around ESG rating divergence. A rigorous mathematical model could be introduced to formalize the problem of ESG rating inconsistency. E.g., let \( R_i^j \) denote the ESG rating assigned to firm \( i \) by provider \( j \). The divergence between two providers \( j \) and \( k \) for a given firm \( i \) could be measured through a divergence metric, such as the Euclidean norm:

 \[

 D_{ik} = \sqrt{\sum_{j=1}^{N} (R_i^j - R_i^k)^2}

 \]

 where \( N \) represents the total number of ESG rating providers under consideration. Analogously, as an alternative to the latter, one could consider a Mahalanobis distance approach to account for the covariance structure between ratings.

 Moreover, theoretical discussions on PCA, Bayesian aggregation, and ML-methods provide valuable insights, but their effectiveness in synthesizing ESG ratings remains an open question. An empirical study could be designed in which multiple ESG rating datasets are aggregated using different methodologies, and the resulting aggregated scores are evaluated against financial market outcomes such as credit spreads or stock returns. Specifically, given a set of ESG ratings \( R_{i}^j \), an aggregated ESG score \( S_i \) could be constructed as a linear combination:

 \[

 S_i = \sum_{j=1}^{N} w_j R_i^j

 \]

 where \( w_j \) are weights derived through various optimization approaches, such as Bayesian updating or regression models that maximize correlation with an independent measure of firm sustainability.

 It seems to the underwritten that the lack of such a discussion also weakens the practical applicability of the study. Indeed, ML-techniques such as XGBoost, while powerful in their predictive capabilities, often suffer from low interpretability, making it difficult for investors to understand the underlying structure of the aggregated ESG scores. A possible solution could be to consider SHAP (SHapley Additive exPlanations) values or partial dependence plots to dissect the contribution of individual ESG components to the final score.

 Let me also consider that the divergence of ESG ratings can be examined using statistical measures of dispersion, e.g. (standard deviation)

 \[

 \sigma_i = \sqrt{\frac{1}{N} \sum_{j=1}^{N} (R_i^j - \bar{R}_i)^2}

 \]

 where \( \bar{R}_i \) is the mean ESG rating for firm \( i \), provides an indication of variability. If the standard deviation is high, it implies substantial disagreement among providers. Another approach is to use clustering techniques, such as k-means or hierarchical clustering, to group firms based on rating similarity, revealing whether discrepancies arise due to fundamental methodological differences among rating agencies.

 Analogously, a regression-based approach could also be employed to quantify the determinants of ESG rating divergence. A mixed-effects model of the form:

 \[

 D_{ij} = \alpha + \beta_1 X_{i} + \beta_2 Y_{j} + \epsilon_{ij}

 \]

 where \( X_{i} \) represents firm-level characteristics, and \( Y_{j} \) represents rating-provider-specific attributes, e.g., methodology used and data sources, which could identify the primary drivers of rating inconsistencies.

 Summing up, the submitted paper provides a critical review of ESG rating divergence and aggregation methods, yet it could benefit from a more rigorous theoretical and empirical foundation. It would also be interesting if the Authors could better underline/propose possible future research aiming at, e.g., developing hybrid models that integrate statistical, Bayesian, and ML-approaches while maintaining interpretability. Moreover, the relationship between ESG scores and financial performance should be further explored through dynamic asset pricing models that incorporate ESG factors as state variables.

Are all factual statements correct and adequately supported by citations?

Yes

Is the review written in accessible language?

Yes

Are the conclusions drawn appropriate in the context of the current research literature?

Yes

Is the topic of the review discussed comprehensively in the context of the current literature?

Yes

Reviewer Expertise:

Stochastic analysis and applications, financial arguments included. Machine learning and application to time series forecasting, LLM-based models, calibration methodologies.

I confirm that I have read this submission and believe that I have an appropriate level of expertise to confirm that it is of an acceptable scientific standard, however I have significant reservations, as outlined above.

# Response

**Published:** 2025-06-06

**Authors:** Alessandra Tanda

**Affiliations:** Universita degli Studi di Pavia Dipartimento di Scienze Economiche e Aziendali, Pavia, Lombardia, Italy

We thank the reviewer for his useful comments and suggestions. Below we provide a response to his remarks.   

**>Response:** we thank the reviewer for his comments and suggestions. In the revised version, we have defined the most used metrics in the literature regarding the divergence of ESG scores. In particular, the works analysed in our study express divergence in terms of low pairwise correlations between the ESG ratings of different providers. As ESG scores are usually continuous, the most natural choice to model their correlation is the Pearson correlation coefficient. However, correlation can be also calculated through rank-based measures such as the Spearman coefficient. The latter is used by Billio et al. (2021), together with mean absolute error and the percentage of observed agreement among the considered rating providers.  Correlation is not only employed to measure convergence between the original ESG scores - to show the empirical relevance of the topic of divergence - but can also be included, in the next step of aggregating the scores, to check to what extent the results of the aggregation obtained with different methods are similar (see Bissoondoyal-Bheenick et al., 2024). Concerning the evaluation of the presented methods in producing aggregated scores able to effectively predict the ESG profile of companies, we have first defined the most natural mathematical formulation for an aggregate ESG indicator, i.e. a weighted average of different scores, that can be defined according to an expert-based approach or estimated by data (Berg et al, 2022; Agosto et al., 2023a; Agosto et al., 2023b; Bissoondoyal-Bheenick et al., 2024). In the revised version we have also discussed how difficult it is to measure and compare the performance of ESG aggregated indicators due to the absence of a target variable. Indeed, differently from credit scoring models, which are validated using the actual incidence of defaults, the ESG behaviour cannot be easily and objectively summarised by an observed variable. Indeed, some works in the literature use unsupervised techniques such as PCA ( Bissoondoyal-Bheenick et al., 2024; Gucciardi et al., 2024), while others rely on the relationship between ESG and financial performance (Billio et al., 2021; Gai et al., 2023) or credit worthiness measured by agency ratings (Agosto et al., 2023a; Agosto et al., 2023b). As pointed out by the reviewer and now discussed in the revised version, the unavailability of an homogeneous and easily implementable testing framework represents a limitation of the existing ESG aggregation methodologies. Applying cluster analysis to the proposed ESG scores and identifying common financial and strategic features in companies belonging to the same clusters is now mentioned among the aspects that deserve further research, together with a more in-depth analysis of the topic of explainability of the weights attributed to different scores. Regarding this last aspect, we have emphasized how many machine learning techniques not explainable by design, such as XGBOOST, do not necessarily lead to a gain in predictive accuracy that justifies the use of a computationally expensive AI method like Shapley values, as discussed by Agosto et al. 2023a.   

# Reviewer report: Reviewer response for version 1

**DOI:** 10.21956/openreseurope.20823.r50969

**Published:** 2025-02-22

**Authors:** Vincenzo Capizzi

**Affiliations:** 1 University of Piemonte Orientale, Novara, Italy

The paper reviews the existing literature on the divergence of Environmental, Social, and Governance (ESG) ratings and explores various aggregation methods. It highlights the challenges posed by inconsistent ESG ratings and their implications for investment decisions. The major finding of the analysis is the persistence of significant gaps in understanding how to effectively consolidate ESG information to create a dependable sustainability indicator.

 Overall, the paper is truly interesting in that addresses an actual research topic within the reference extant literature. The research design is truly original and rigorous

 However, there are some limitations in the current draft and, hence, here below I provide some guidelines to further enhance the quality of the manuscript.

- First, the Author might consider improving the introductory section, trying to better clarify the motivation behind their research and the source of originality provided .
- Second, as far as the literature review is concerned, the Authors might explain how they selected the investigated contributions.
- Furthermore, the literature selection seems not updated: try to cite all relevant and recent papers on the same topic.

Finally, the writing style might be improved

 Good luck!

Are all factual statements correct and adequately supported by citations?

Yes

Is the review written in accessible language?

Yes

Are the conclusions drawn appropriate in the context of the current research literature?

Yes

Is the topic of the review discussed comprehensively in the context of the current literature?

Yes

Reviewer Expertise:

Finance, Entrepreneurial Finance, ESG ratings, sustainable finance,

I confirm that I have read this submission and believe that I have an appropriate level of expertise to confirm that it is of an acceptable scientific standard.

# Response

**Published:** 2025-06-06

**Authors:** Alessandra Tanda

**Affiliations:** Universita degli Studi di Pavia Dipartimento di Scienze Economiche e Aziendali, Pavia, Lombardia, Italy

Response to reviewers for the paper Divergence and aggregation of ESG ratings:A survey

 Reviewer 1 : We thank the reviewer for his comments and suggestions. Below we have provided a response to his very useful comments. 

 First, the Author might consider improving the introductory section, trying to better clarify the motivation behind their research and the source of originality provided >

 Response: we thank the reviewer for his comments and suggestions. We have rewritten the Introduction and different sections of the paper to integrate the suggestions and clarify the motivation and the contribution of the paper.

 Second, as far as the literature review is concerned, the Authors might explain how they selected the investigated contributions  >

 Response: we appreciate reviewer's suggestion. In the new version we have included a short section describing the process employed to select the papers for our review. 

 Furthermore, the literature selection seems not updated: try to cite all relevant and recent papers on the same topic. >

 Response: In the new version we have updated the literature review and included more recent papers.
